# Obstructed Hemivagina with Ipsilateral Renal Anomaly: Radiological Diagnosis, Outcome of Surgical Management and Literature Review

**DOI:** 10.3390/diagnostics16040537

**Published:** 2026-02-11

**Authors:** Hanadi Bakhsh, Anas Hamdoun

**Affiliations:** 1 Obstetrics and Gynecology Department, College of Medicine, Princess Nourah bint Abdulrahman University, Riyadh 11564, Saudi Arabia; 2 Department of Obstetrics and Gynecology, King Abdullah Bin Abdulaziz University Hospital, Princess Nourah bint Abdulrahman University, Riyadh 13412, Saudi Arabia; 3 Medical Imaging Department, King Abdullah Bin Abdulaziz University Hospital, Princess Nourah bint Abdulrahman University, Riyadh 13412, Saudi Arabia; aohamdoun@kaauh.edu.sa

**Keywords:** OHVIRA syndrome, obstructed hemivagina, uterine didelphys, renal agenesis, pelvic MRI, adolescent gynecology, Müllerian duct anomaly, vaginal septum resection

## Abstract

**Background and Clinical Significance:** Obstructed hemivagina and ipsilateral renal anomaly (OHVIRA) syndrome is a rare Müllerian duct anomaly that is frequently misdiagnosed as primary dysmenorrhea, resulting in delayed intervention and preventable complications such as hematocolpos, pelvic infection, adnexal damage, and endometriosis. Accurate early diagnosis relies on imaging, yet the complementary roles of ultrasound and magnetic resonance imaging (MRI) remain underemphasized in routine clinical pathways. This study highlights contemporary diagnostic strategies and surgical outcomes to support timely, fertility-preserving management. **Case Presentation:** We report a retrospective case series of four adolescent patients diagnosed with OHVIRA syndrome between 2020 and 2025. All patients underwent pelvic ultrasound as an initial diagnostic modality, followed by MRI for definitive anatomical characterization. Imaging findings, surgical management, and clinical outcomes are presented and compared with a systematically screened review of recent literature. One case represents a rare prepubertal variant with ectopic ureteral communication. Surgical management primarily involved single-stage hemivaginal septal resection, with individualized approaches based on anatomy and clinical presentation. **Conclusions:** Ultrasound remains an effective first-line screening tool for suspected obstructive Müllerian anomalies, while MRI is essential for definitive diagnosis, identification of associated urinary tract variants, and surgical planning. Early MRI-guided intervention enables symptom resolution and may reduce long-term reproductive morbidity. Standardized longitudinal follow-up into reproductive age is required to confirm fertility outcomes and optimize evidence-based management of OHVIRA syndrome.

## 1. Introduction

OHVIRA syndrome (obstructed hemivagina and ipsilateral renal anomaly), historically referred to as Herlyn–Werner–Wunderlich syndrome, is a rare congenital malformation characterised by the classic triad of uterine didelphys, unilateral obstructed hemivagina, and ipsilateral renal anomaly, most commonly renal agenesis [1]. OHVIRA represents a complex developmental disorder arising from concurrent abnormalities of the Müllerian (paramesonephric) and Wolffian (mesonephric) ducts and typically manifests during adolescence after menarche, when functional endometrium leads to progressive outflow obstruction and blood accumulation in the obstructed vaginal and uterine compartments [2].

The embryological basis of OHVIRA lies in the close developmental interdependence between the Müllerian and Wolffian duct systems. During early embryogenesis, the Wolffian ducts play a critical inductive role in the normal development and fusion of the Müllerian ducts [3]. Failure of normal Wolffian duct development on one side can result in renal agenesis and simultaneously impair ipsilateral Müllerian duct fusion and canalization, leading to uterine didelphys and an obstructed hemivagina [4]. This shared embryological origin explains the consistent association between genital tract duplication anomalies and ipsilateral urinary tract abnormalities in OHVIRA, underscoring the importance of evaluating the urinary system whenever a Müllerian anomaly is suspected [5,6].

Despite its well-described anatomical triad, OHVIRA is frequently underdiagnosed or diagnosed late, particularly because many affected adolescents initially experience an apparently normal menarche and regular menstrual cycles [7,8]. Early symptoms are often nonspecific and may include cyclic pelvic or lower abdominal pain, urinary or bowel symptoms, dysmenorrhea, or progressively light menstrual flow due to partial decompression of the obstructed compartment [9]. As a result, the condition may be misinterpreted as primary dysmenorrhea, functional ovarian cysts, or nonspecific pelvic pain, leading to delayed referral and imaging. Diagnostic delay has important clinical consequences, including hematocolpos, hematometra, hematosalpinx, pelvic infection, endometriosis, tubal torsion, and future fertility impairment [10,11].

The severity and nature of complications are closely related to the duration of obstruction and the presence of retrograde menstruation. Prolonged retention of menstrual blood can lead to distension of the vagina and uterus, secondary involvement of the fallopian tube, and inflammatory sequelae within the pelvis [12,13]. Several recent reports have highlighted presentations complicated by pyosalpinx, tubo-ovarian abscess, extensive endometriosis, or acute abdominal emergencies such as adnexal torsion, emphasising the morbidity associated with delayed recognition of the syndrome [14,15]. In rare cases, OHVIRA may be detected in the prepubertal period or during pregnancy, often in association with infection or urinary tract complications, further illustrating the heterogeneous clinical spectrum of this condition [16,17].

Imaging plays a central role in the diagnosis of OHVIRA. Pelvic ultrasound is typically the first-line imaging modality due to its availability and non-invasiveness and may demonstrate a distended, fluid-filled vagina or uterus suggestive of hematocolpos or hematometrocolpos [18]. However, ultrasound is limited in its ability to fully delineate complex Müllerian anatomy, accurately classify uterine anomalies, and identify associated complications or urinary tract abnormalities [19]. Consequently, ultrasound findings may be inconclusive or misinterpreted, particularly in patients with atypical anatomy or early disease.

Magnetic resonance imaging (MRI) is widely regarded as the imaging modality of choice for OHVIRA. MRI provides superior soft-tissue contrast and multiplanar capability, allowing precise characterisation of uterine morphology, vaginal septa, the level and side of obstruction, and associated findings such as hematosalpinx, endometriosis, or pelvic inflammatory changes [20]. In addition, MRI enables comprehensive assessment of the urinary tract, including confirmation of renal agenesis, ectopic ureteral insertion, or other associated anomalies, without exposure to ionising radiation [21]. Accurate preoperative MRI assessment is critical for surgical planning and for avoiding incomplete correction or postoperative recurrence due to inadequate septal resection [22].

Surgical management of OHVIRA aims to relieve the outflow obstruction while preserving reproductive potential. Resection of the obstructing vaginal septum with marsupialization is considered the definitive treatment and is associated with excellent symptom relief when performed adequately [23]. Laparoscopy is often employed concurrently to assess pelvic anatomy, manage complications such as hematosalpinx or endometriosis, and address acute conditions including tubal torsion or infection [24]. Recent literature has demonstrated favourable outcomes with timely surgical intervention, whereas delayed diagnosis is associated with higher rates of secondary procedures and adnexal damage [25].

Given the rarity of OHVIRA and the wide variability in clinical presentation, case series remain valuable for expanding clinical awareness, illustrating diagnostic challenges, and highlighting the role of imaging and tailored surgical management. The present case series describes four patients with OHVIRA presenting with diverse symptoms, radiological findings, and complications, including adolescent and prepubertal presentations. By integrating detailed radiological assessment, surgical outcomes, and a narrative review of recent literature, this study aims to emphasise the pivotal role of MRI in diagnosis, demonstrate the spectrum of disease manifestations, and reinforce the importance of early recognition and appropriate surgical intervention to minimise morbidity and optimise outcomes [26].

## 2. Materials and Methods

This study was conducted as a retrospective case series describing the radiological characteristics, surgical management, and clinical outcomes of patients diagnosed with obstructed hemivagina with ipsilateral renal anomaly (OHVIRA) syndrome. A retrospective design was selected to allow comprehensive review of imaging findings, operative records, and follow-up data in patients managed for this rare congenital condition. The study follows accepted methodological standards for case series reporting and is intended to provide descriptive and comparative insights rather than inferential statistical analysis.

### 2.1. Study Setting and Patients

The case series was conducted at King Abdullah bin Abdulaziz University Hospital with specialised services in pediatric and adolescent gynecology, radiology, and minimally invasive surgery. Medical records from patients managed between January 2021 and October 2024 were reviewed.

Patients were eligible for inclusion if they met all of the following criteria:

1. A confirmed diagnosis of uterine didelphys with unilateral obstructed hemivagina on imaging;
2. An associated ipsilateral renal anomaly, including renal agenesis, solitary kidney, or ectopic ureteral abnormality;
3. Availability of preoperative imaging studies (ultrasound and/or MRI pelvis);
4. Clinical management at the study institution, including surgical intervention or documented follow-up.

Patients with incomplete imaging data or without confirmed ipsilateral renal involvement were excluded. Four patients fulfilled the inclusion criteria and were included in the final analysis. Clinical data were extracted from electronic medical records and included age at presentation, presenting symptoms, menstrual history, physical examination findings, imaging results, operative reports, and postoperative outcomes.

### 2.2. Imaging Protocol

All patients underwent pelvic imaging as part of their diagnostic workup. Transabdominal pelvic ultrasound was used as the initial imaging modality in all cases, given its accessibility and suitability for adolescent patients. Ultrasound examinations assessed the presence of vaginal or uterine distension, fluid characteristics suggestive of hematocolpos or hematometrocolpos, uterine morphology, adnexal structures, and urinary tract anatomy, including renal presence and pelvicalyceal dilatation.

Magnetic resonance imaging (MRI) of the pelvis was performed in all patients to further characterise Müllerian anatomy and associated abnormalities. MRI studies included multiplanar T1- and T2-weighted sequences, with axial, sagittal, and coronal planes, and additional fat-suppressed sequences when indicated. MRI evaluation focused on:

- Classification of uterine morphology (e.g., uterine didelphys);
- Identification and side of vaginal obstruction;
- Extent of vaginal, uterine, and tubal distension;
- Signal characteristics of retained contents (blood, fluid, or infected material);
- Assessment of associated pelvic complications, including hematosalpinx, adnexal torsion, inflammatory changes, or endometriotic deposits;
- Evaluation of urinary tract anomalies, including renal agenesis, solitary kidney, ectopic ureter, or ureteral dilatation.

MRI findings were reviewed collaboratively by radiologists and treating clinicians to guide surgical planning and to determine the need for concurrent laparoscopy.

### 2.3. Surgical Management

All surgically managed patients underwent resection of the obstructing hemivaginal septum, which constituted the definitive treatment for restoring vaginal outflow. The procedures were performed under general anesthesia with careful attention to preserving vaginal anatomy and ensuring long-term patency.

The surgical approach involved identification of the obstructed hemivagina, followed by an appropriately sized incision of the vaginal septum to allow complete drainage of retained menstrual blood or purulent material when present. Particular emphasis was placed on creating a sufficiently wide opening to reduce the risk of restenosis. The edges of the septal incision were marsupialized using interrupted sutures, ensuring continuous communication between the previously obstructed and non-obstructed vaginal lumens.

Diagnostic and therapeutic laparoscopy was performed concurrently in selected patients based on imaging findings or intraoperative assessment. Laparoscopy allowed direct visualization of uterine morphology, adnexal structures, and pelvic cavity, and facilitated management of associated complications. In cases with hematosalpinx, adnexal torsion, or severe inflammatory changes, laparoscopic intervention included drainage, adhesiolysis, detorsion, or salpingectomy when tubal viability was compromised. The decision to perform adnexal surgery was based on intraoperative findings and tissue viability.

Postoperative management included analgesia, antibiotic therapy when infection was suspected or confirmed, and hormonal suppression therapy in selected cases with endometriotic disease, as determined by the treating gynecology team.

### 2.4. Follow-Up

All patients were followed clinically after surgery to assess symptom resolution, menstrual flow, and potential complications. Follow-up schedules were individualized but generally included outpatient reviews within the first few weeks after surgery and subsequent visits over the following months.

Follow-up pelvic ultrasound was performed to confirm resolution of hematocolpos or other fluid collections, assess vaginal patency, and evaluate uterine and adnexal structures. Clinical outcomes assessed during follow-up included improvement in pelvic pain, normalization of menstrual flow, absence of recurrent vaginal obstruction, and resolution of urinary or bowel symptoms. Patients without surgical intervention or those lost to follow-up were documented accordingly.

### 2.5. Narrative Literature Review

To contextualize the findings of the present case series and to situate them within the evolving body of evidence on obstructed hemivagina with ipsilateral renal anomaly (OHVIRA) syndrome, a narrative literature review was conducted. A narrative approach was considered most appropriate given the rarity of the condition, the predominance of case reports and small case series in the literature, and the heterogeneity of reported clinical presentations, imaging strategies, and surgical techniques.

Electronic searches were performed in PubMed (MEDLINE), Scopus, and Google Scholar to identify studies published between January 2020 and December 2025. The full database-specific search strategies (including Boolean operators and keywords/MeSH terms) are provided in Table 1.

Eligibility criteria were predefined. Inclusion criteria were: (1) English-language publications (January 2020–December 2025); (2) reports describing OHVIRA syndrome (obstructed hemivagina with ipsilateral renal/urinary tract anomaly), including variants reported under “Herlyn–Werner–Wunderlich syndrome”; and (3) articles providing clinically relevant information on imaging (ultrasound and/or MRI), surgical management, and/or outcomes. Eligible designs included case reports, case series, imaging studies, and reviews. Exclusion criteria were: (1) Müllerian anomalies without ipsilateral renal/urinary tract involvement; (2) articles lacking sufficient clinical/imaging detail (e.g., conference abstracts without full text); (3) non-human studies; and (4) duplicate reports of the same cases without additional data.

All retrieved records were screened by title/abstract for relevance, followed by full-text assessment for eligibility. Reference lists of included articles were also screened to identify additional relevant publications.

Rather than attempting quantitative synthesis, the extracted literature was analyzed qualitatively. Key themes were identified, including variability in age at presentation, spectrum of symptoms, imaging pitfalls, MRI diagnostic criteria, association with pelvic complications such as endometriosis or infection, and differences in surgical approach. Particular attention was paid to studies published in the last two years that reported surgical outcomes, long-term patency, fertility implications, and complications associated with delayed diagnosis, as these aspects were directly relevant to the cases presented in this series.

The findings from the reviewed literature were synthesized narratively and compared with the clinical and radiological features observed in the current cohort. This comparative approach allowed identification of shared patterns as well as distinctive features, such as complicated presentations involving tubal torsion, pyosalpinx, or prepubertal diagnosis with ectopic ureteral communication. The literature review also informed the discussion of best practices in imaging-based diagnosis, surgical timing, and operative technique, particularly the importance of adequate septal resection and marsupialization to prevent restenosis.

## 3. Results

### 3.1. Overview of the Case Series

Four female patients diagnosed with obstructed hemivagina with ipsilateral renal anomaly (OHVIRA) syndrome were included in this case series. The patients’ ages ranged from 11 to 16 years, encompassing both adolescent and prepubertal presentations. All cases demonstrated uterine didelphys associated with unilateral vaginal obstruction and an ipsilateral renal anomaly, most commonly renal agenesis. Presenting symptoms varied and included cyclic or progressive lower abdominal pain, urinary symptoms, amenorrhea or altered menstrual flow, abdominal mass, and, in complicated cases, fever and foul-smelling vaginal discharge.

Pelvic ultrasound was the initial imaging modality in all cases and consistently demonstrated a distended, fluid-filled vagina suggestive of hematocolpos. MRI pelvis was subsequently performed in all patients and provided definitive anatomical characterization, allowing accurate diagnosis of uterine morphology, localization of the obstructed hemivagina, assessment of retained contents, and identification of associated pelvic and urinary tract abnormalities. Surgical management was individualized based on imaging findings and clinical presentation. A summary of demographic data, imaging findings, surgical interventions, and outcomes for all four cases is presented in Table 2 and Table 3.

### 3.2. Individual Case Presentations

**Case 1**

A 16-year-old girl was referred for further management of suspected OHVIRA syndrome after initial treatment at another institution. She had initially presented with fever and left iliac fossa pain. Contrast-enhanced CT performed externally demonstrated a large multiloculated cystic pelvic mass involving the left adnexa, measuring approximately 12.2 × 12.2 × 8.2 cm, associated with inflammatory changes and peritoneal fat stranding.

The patient underwent laparoscopic drainage of a left pyosalpinx, with extensive adhesiolysis. Intraoperative findings included inflammatory adhesions involving the omentum and left adnexa, with pyosalpinx affecting approximately three-quarters of the left tube. Both ovaries were normal. Postoperatively, she received intravenous antibiotics followed by oral doxycycline.

**Figure 1:**
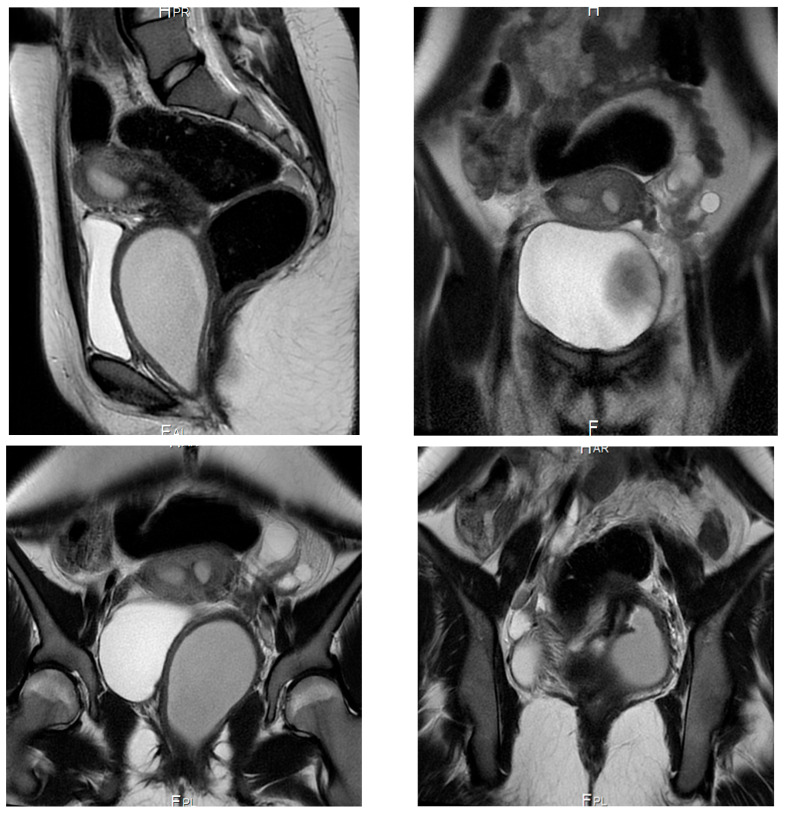

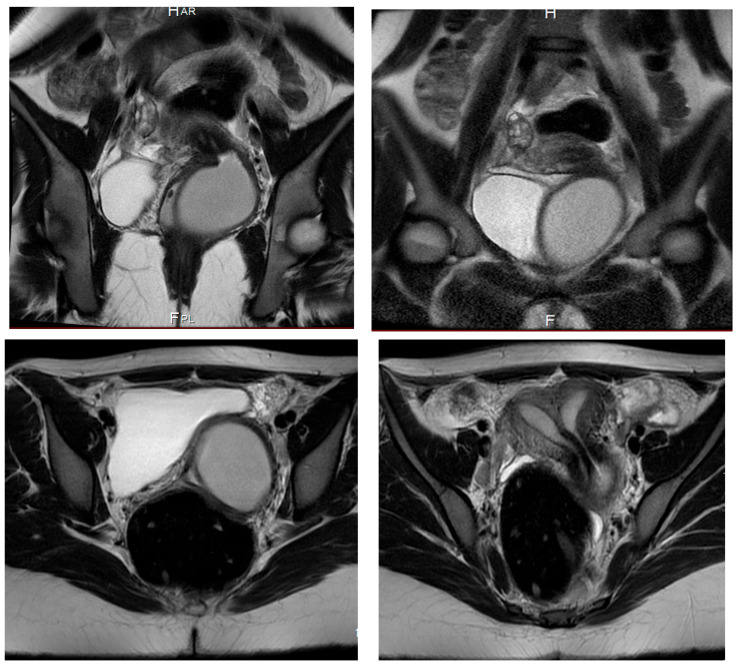
MRI pelvis (external study) in Case 1 demonstrating uterine didelphys with two cervices and a dilated left hemivagina filled with fluid due to distal vaginal obstruction. Absent left kidney is also noted.

Subsequent MRI pelvis performed externally demonstrated uterine didelphys with two cervices, a dilated obstructed left hemivagina filled with fluid, and absence of the left kidney (Figure 1). On presentation to the study institution, the patient complained of lower abdominal pain and foul-smelling vaginal discharge. Menstrual history revealed menarche at age 12 years, regular cycles, but recent reduction in menstrual flow and postmenstrual pain.

Based on imaging findings, the patient underwent left hemivaginal septal resection with marsupialization, resulting in drainage of retained malodorous blood. She was discharged on oral doxycycline. Follow-up demonstrated normal menstrual flow, resolution of discharge, and significant pain reduction, with maintained vaginal patency.

**Case 2**

A 15-year-old adolescent girl presented to the emergency department with severe lower abdominal pain that had progressively worsened over a two-month period. The pain was associated with difficulty in urination and defecation, without fever, nausea, or vomiting. Menarche had occurred at age 13 years, with initially regular menstrual cycles lasting 4–5 days. Over the preceding two years, the patient reported progressively lighter menstrual flow accompanied by recurrent episodes of lower abdominal pain following menstruation.

Physical examination revealed normal secondary sexual characteristics. Abdominal examination demonstrated a distended, non-tender abdomen with a mobile, globular mass extending above the umbilicus, corresponding to approximately 22 weeks’ gestational size.

**Figure 2:**
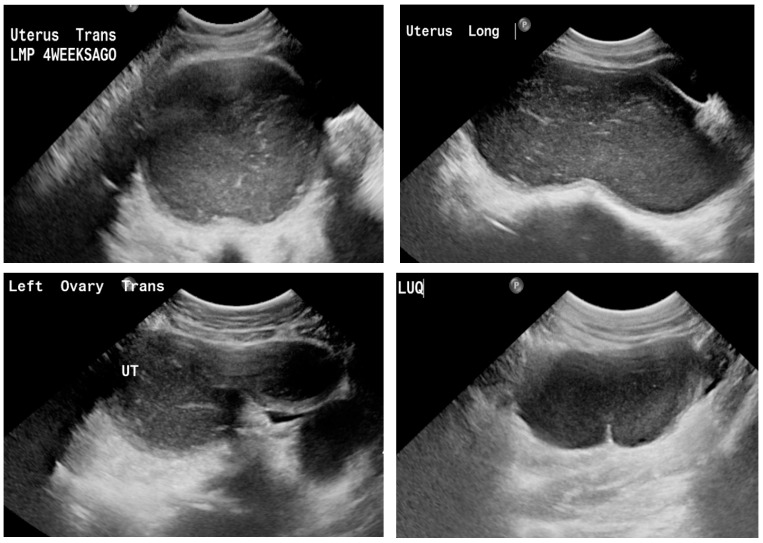
Transabdominal pelvic ultrasound (axial and longitudinal views) demonstrating a markedly distended fluid-filled vagina consistent with hematocolpos in a 15-year-old girl (Case 2).

**Figure 3:**
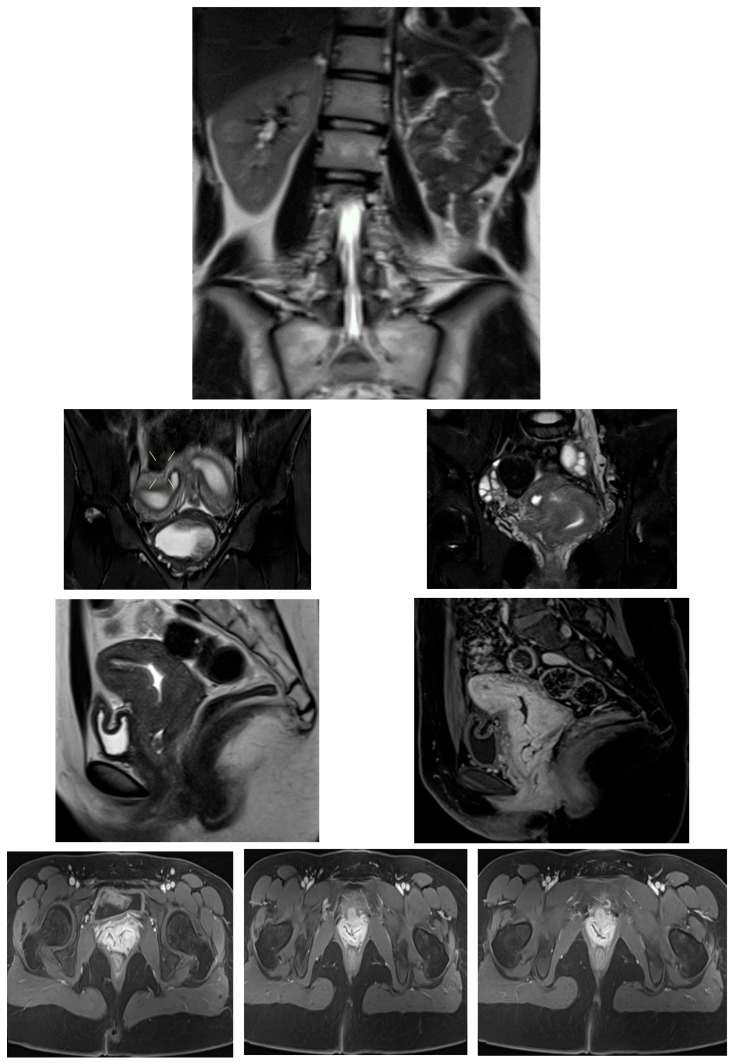
MRI pelvis in Case 2 demonstrating uterine didelphys with left distal hemivaginal obstruction and a large blood-filled elongated vagina/hematocolpos forming a pelvi-abdominal cystic mass. Absence of the left kidney is noted, consistent with OHVIRA syndrome.

Transabdominal pelvic ultrasound demonstrated a markedly distended, fluid-filled vagina, consistent with hematocolpos (Figure 2). Subsequent MRI pelvis revealed an elongated, blood-filled vagina forming a large pelvi-abdominal cystic mass, compressing the urinary bladder anteriorly and the rectum posteriorly (Figure 3). The uterus was displaced superiorly and demonstrated features consistent with uterine didelphys. An elongated blood-filled structure consistent with left hematosalpinx was also identified. Both ovaries appeared normal. The left kidney was absent, with mild dilatation of the right renal pelvis and ureter. No enhancing pelvic masses were identified.

**Figure 4:**
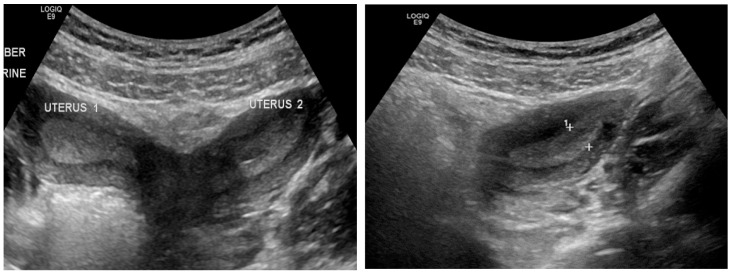
Postoperative follow-up transabdominal ultrasound in Case 2 demonstrating complete resolution of hematocolpos with restoration of normal vaginal anatomy following hemivaginal septal resection.

The patient underwent left hemivaginal septal resection with marsupialization, resulting in immediate drainage of retained old menstrual blood. Diagnostic laparoscopy demonstrated a markedly distended and edematous left fallopian tube with two complete torsions around the ovary. Despite decompression, tubal viability was poor, and left salpingectomy was performed. Postoperative recovery was uneventful. Follow-up ultrasound demonstrated a normal vaginal cavity with complete resolution of hematocolpos (Figure 4), and the patient reported significant symptom improvement.

**Case 3**

A 13-year-old girl presented with acute right lower quadrant abdominal pain associated with difficulty passing urine. Menarche had occurred at age 11 years, with previously regular menstrual cycles lasting 7–8 days. Over the preceding six months, she experienced secondary amenorrhea accompanied by monthly episodes of cyclic lower abdominal pain.

**Figure 5:**
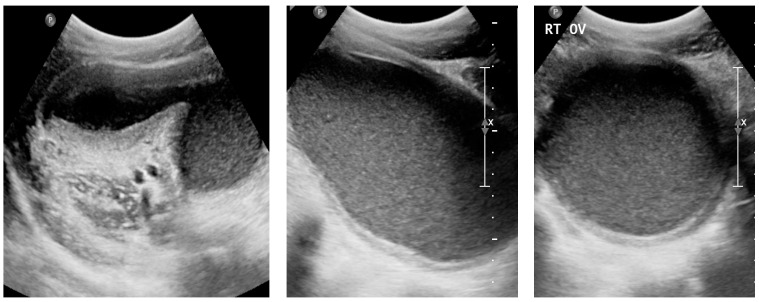
Pelvic ultrasound in a 13-year-old patient (Case 3) showing a markedly distended fluid-filled vagina consistent with hematocolpos secondary to obstructed right hemivagina.

Physical examination showed normal pubertal development. Abdominal examination revealed a non-tender, mobile pelvic mass extending to approximately 20 weeks’ gestational size. Initial pelvic ultrasound demonstrated a markedly distended fluid-filled vagina, consistent with hematocolpos (Figure 5).

**Figure 6:**
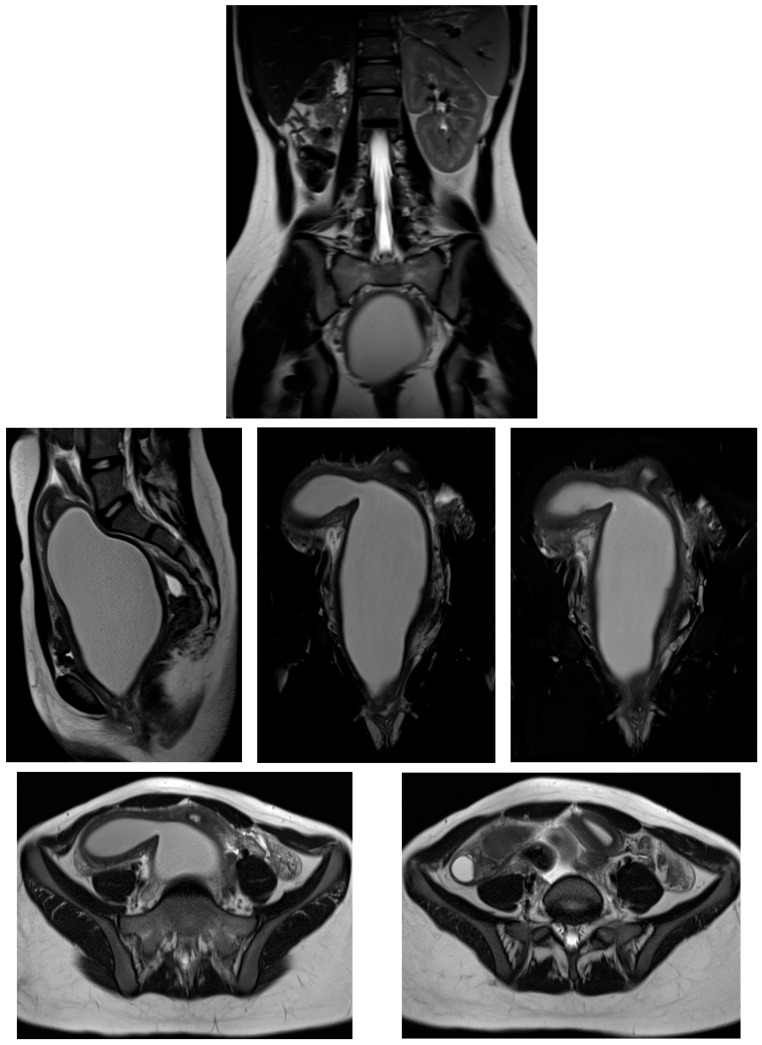

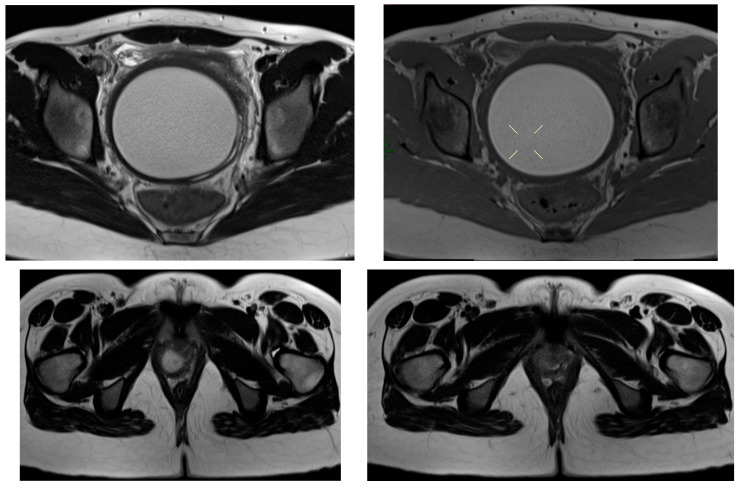
MRI pelvis in Case 3 demonstrating uterine didelphys, obstructed right hemivagina, and right renal agenesis, confirming the diagnosis of OHVIRA (Herlyn–Werner–Wunderlich) syndrome. The left kidney is solitary and normal.

MRI pelvis confirmed a Class III Müllerian duct anomaly (uterine didelphys) with an obstructed right hemivagina and right renal agenesis, consistent with OHVIRA (Herlyn–Werner–Wunderlich) syndrome. The left kidney was solitary and normal (Figure 6).

The patient underwent right hemivaginal septal resection with marsupialization, resulting in drainage of retained menstrual blood. Laparoscopy demonstrated a didelphys uterus with mild distension of the right fallopian tube and scattered endometriotic deposits involving the right tube, omentum, and pelvic sidewalls. Both ovaries appeared normal. Postoperatively, the patient was commenced on dienogest (Visanne) for suppression of endometriosis. Follow-up visits confirmed sustained symptom resolution and maintained vaginal patency.

**Case 4**

An 11-year-old prepubertal girl with a known bicornuate uterus and congenital solitary kidney was followed by pediatric endocrinology for precocious puberty, for which she received gonadotropin-releasing hormone agonist therapy for two years.

**Figure 7:**
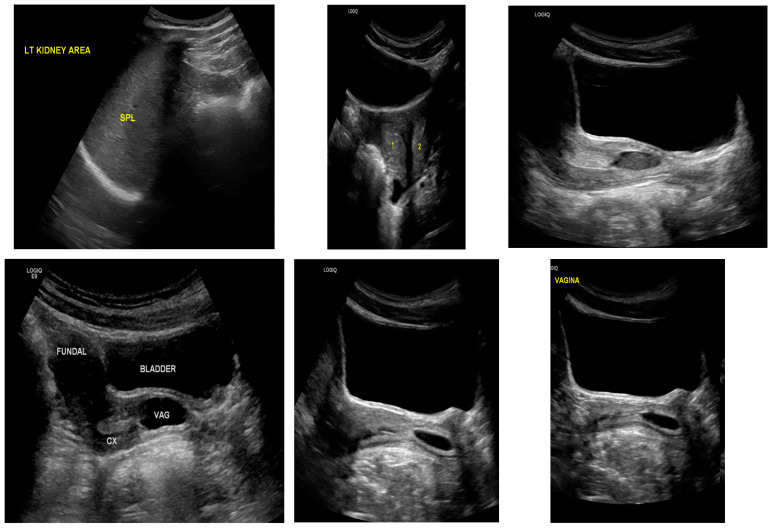
Transabdominal pelvic ultrasound in an 11-year-old prepubertal patient (Case 4) demonstrating cystic structures along the left vaginal wall, without evidence of hematocolpos at the time of examination.

**Figure 8:**
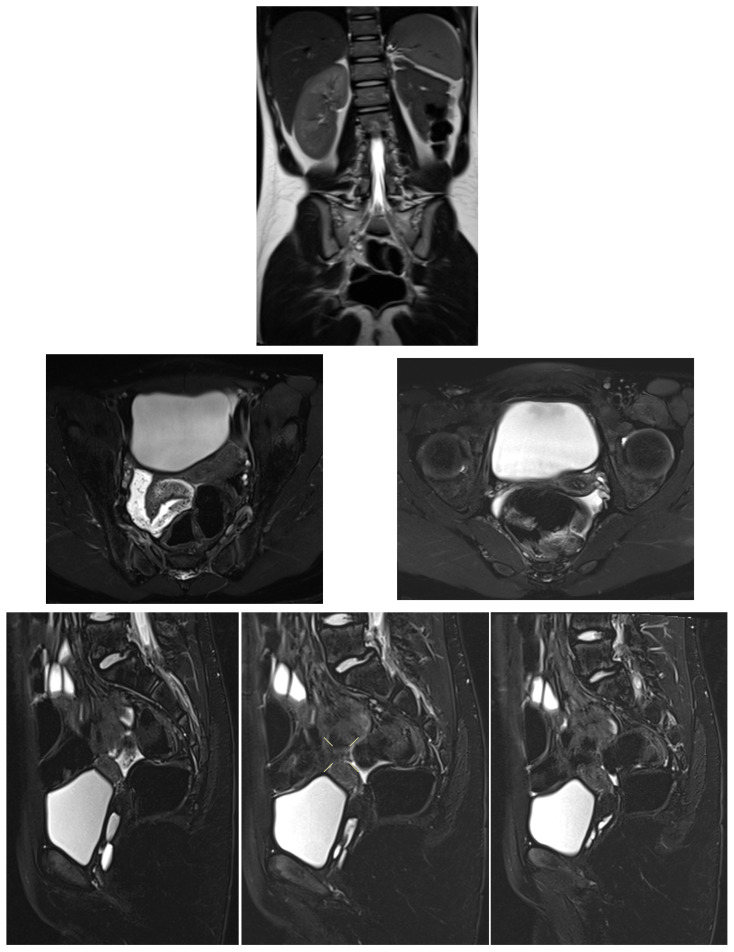

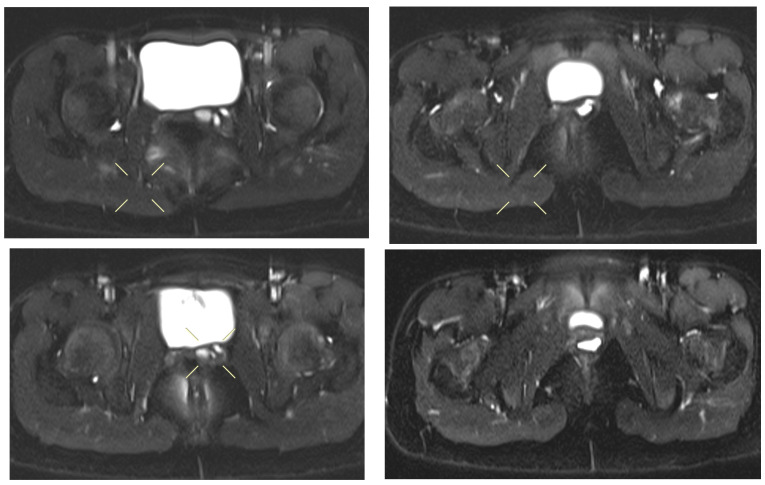
MRI pelvis in Case 4 showing uterine didelphys with an upper vaginal cystic structure communicating with the left uterine horn, resulting in narrowing of the upper vaginal cavity.

**Figure 9:**
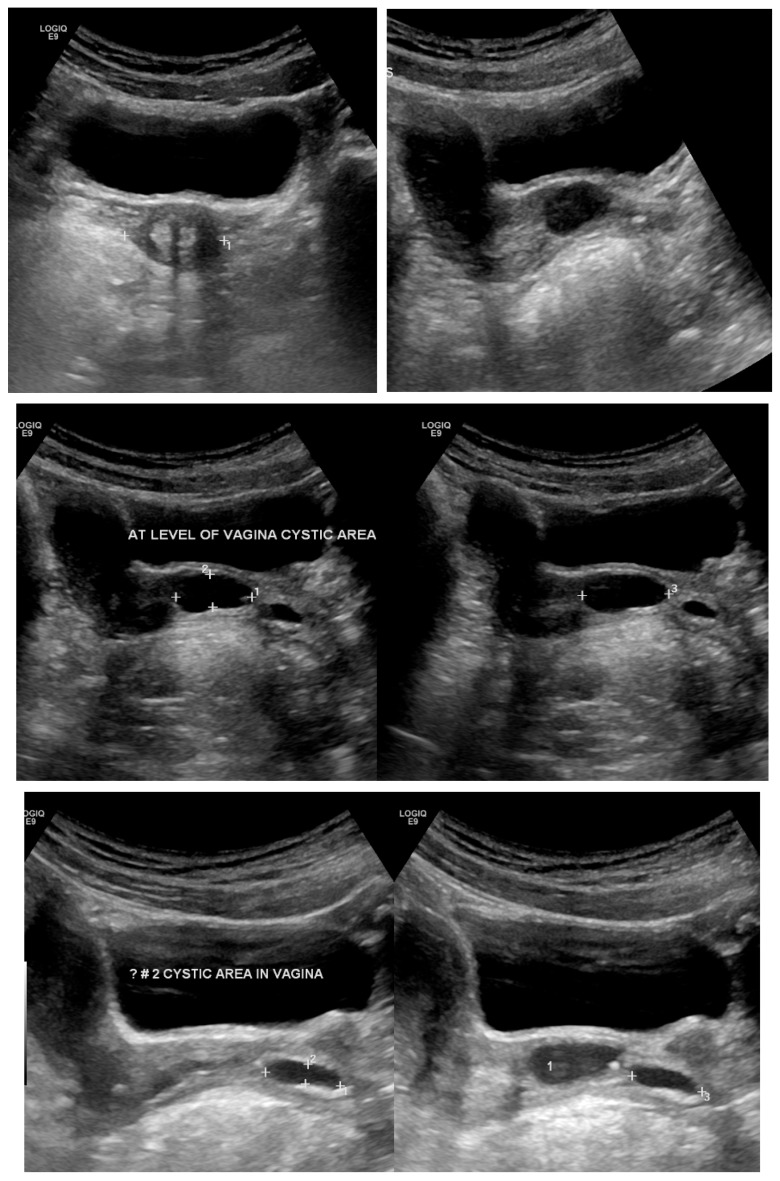
Follow up Transabdominal pelvic ultrasound in Case 4 demonstrating a lower vaginal cystic structure communicating with an ectopic/dysplastic left ureter, associated with congenital absence of the left kidney.

**Figure 10:**
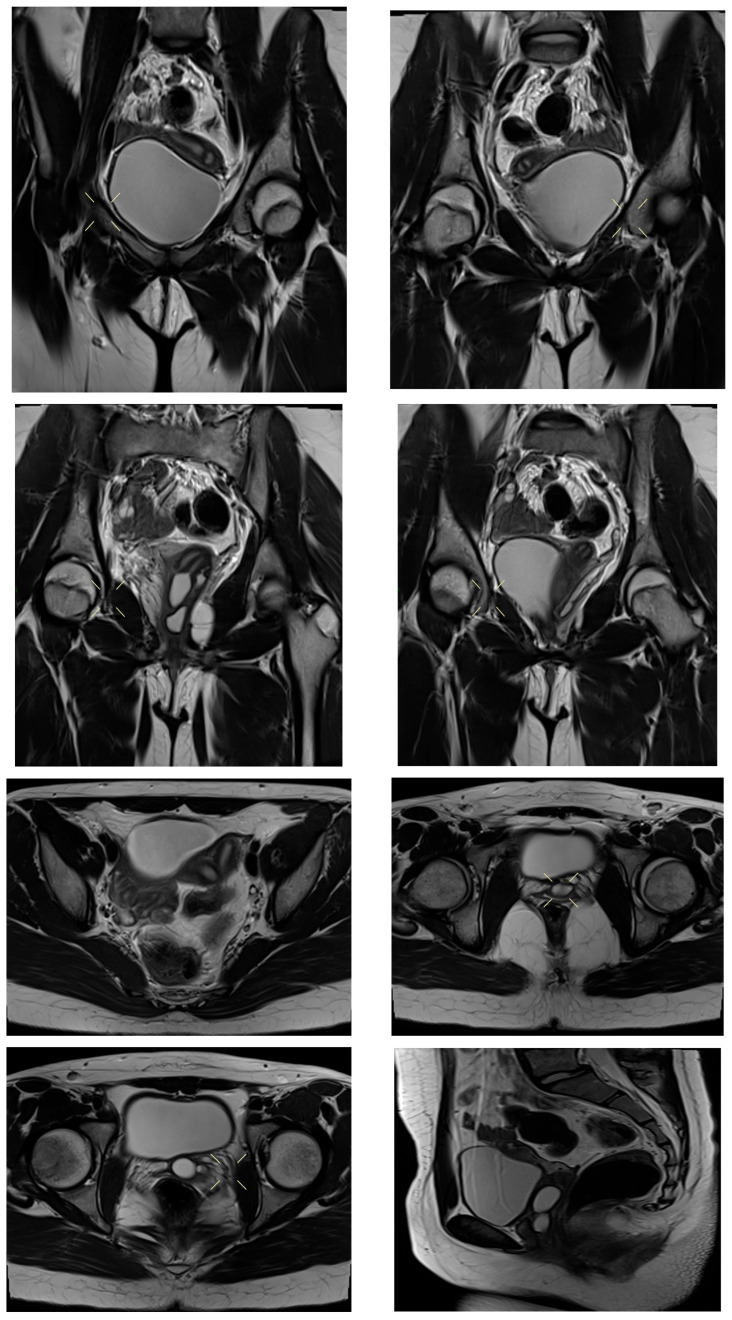
Follow-up MRI pelvis in Case 4 confirming persistence of left-sided vaginal cystic structures with ureteral communication and no evidence of hematocolpos or hematometrocolpos at the time of imaging.

MRI pelvis demonstrated uterine didelphys with two cystic structures along the left anterolateral vaginal wall. The upper cystic structure communicated with the left uterine horn, causing narrowing of the upper vaginal cavity, while the lower cystic structure communicated with an ectopic/dysplastic left ureter (Figure 7, Figure 8, Figure 9 and Figure 10). Both ovaries were present, and no hematocolpos or hematometrocolpos was identified at the time of imaging.

Surgical intervention was planned; however, the patient was subsequently lost to follow-up, and no operative management was performed.

### 3.3. Results of the Narrative Literature Review

The narrative literature review identified a substantial body of evidence describing the clinical presentation, diagnostic pathways, radiological features, surgical management, and outcomes of patients with obstructed hemivagina with ipsilateral renal anomaly (OHVIRA) syndrome (Table 4). Although the condition remains rare, the cumulative literature, dominated by case reports, case series, and a limited number of retrospective cohorts, provides consistent and clinically actionable insights into optimal diagnostic and therapeutic strategies.

Across the reviewed studies, recurring patterns emerged regarding the central role of imaging, the effectiveness of vaginal septal resection, the impact of delayed diagnosis on morbidity, and the breadth of anatomical and clinical variability, particularly in pediatric and urological presentations. Rather than summarizing individual studies in isolation, the findings were synthesized thematically to highlight convergent evidence and to emphasize clinically relevant messages supported by multiple independent publications.

The results of the narrative literature review are therefore presented under four overarching themes, each reflecting a dominant and reproducible outcome reported across the literature. These themes integrate radiological, surgical, and clinical perspectives and provide an evidence-based framework for understanding contemporary OHVIRA management and outcomes.

**1.**  **Diagnostic Approaches: The Complementary Role of Ultrasound and MRI**

Timely and accurate diagnosis of OHVIRA syndrome relies on a combination of clinical suspicion and targeted imaging. Pelvic ultrasound remains the most accessible and commonly used initial modality. In multiple pediatric and adolescent case series, ultrasound effectively identified hematocolpos and uterine duplication when prompted by renal anomalies such as unilateral agenesis [32]. Particularly in centers with skilled sonographers, transabdominal or transperineal ultrasound allowed for early detection of vaginal distension or a duplicated uterine system, facilitating prompt referral for further assessment [33].

Despite its utility, ultrasound has limitations in fully characterizing the complexity of Mullerian anomalies. In this regard, pelvic MRI has emerged as the definitive imaging modality. Across multiple studies, MRI provided superior delineation of uterine configuration, precise identification of the obstructing hemivaginal septum, and clarification of communication between vaginal canals [33]. MRI also proved essential in identifying complications such as hematosalpinx or associated endometriosis, and in preoperative planning, especially in patients with long septal distances or anatomical variants. In fact, in one retrospective review, MRI achieved 100% preoperative diagnostic accuracy in confirming OHVIRA anatomy and guiding single-stage surgical management [33]. These findings underscore the recommended protocol: ultrasound as a first-line screening tool, followed by MRI for comprehensive anatomical assessment and surgical planning.

**2.**  **Surgical Management: Vaginal Septum Resection as a Definitive Solution**

The core therapeutic intervention for OHVIRA syndrome is surgical resection of the obstructing vaginal septum. In nearly all reported cases, this approach resulted in rapid symptom relief and long-term resolution of obstruction. Transvaginal septum excision, often accompanied by marsupialization or vaginoplasty, was the standard technique across diverse patient ages and anatomical variants [31,32]. Even in complex presentations, such as high or oblique septa, or cases with septum length >5 cm, transvaginal resection remained feasible, occasionally augmented by intraoperative ultrasound guidance or temporary stenting to maintain patency [32].

In select cases, particularly when coexistent pathology was suspected, adjunctive laparoscopy played a diagnostic and therapeutic role. For example, authors utilized laparoscopy to treat endometriosis and release adhesions in nearly 20% of their cohort, emphasizing the added benefit of a minimally invasive abdominal approach. Nevertheless, routine laparoscopy is not universally indicated and is reserved for patients with suspected complications or ambiguous anatomy [34]. Importantly, literature consensus supports that vaginal septum resection alone is sufficient in the majority of cases and can be safely performed even in younger patients, provided the anatomy is well defined via imaging [33].

Across studies, surgical outcomes were highly favorable, with minimal rates of re-obstruction, low postoperative morbidity, and successful restoration of normal menstruation. The consistency of these results highlights the curative potential of septal excision and reinforces the importance of early surgical referral upon diagnosis.

**3.**  **Complications of Delayed Diagnosis: Endometriosis, Infection, and Organ Damage**

The literature strongly supports a direct relationship between delayed diagnosis of OHVIRA and the development of gynecologic and urologic complications. Among the most frequently reported sequelae is pelvic endometriosis, likely resulting from chronic retrograde menstruation due to unrelieved obstruction. authorsreported endometriosis in 19% of patients, with the highest incidence (37%) occurring in cases of complete hemivaginal obstruction. Similarly, authors identified endometriosis in 13.6% of patients assessed laparoscopically, suggesting a potential underdiagnosis in others.

Infection is another critical complication. Accumulated blood products in obstructed systems can become secondarily infected, leading to pyocolpos, tubo-ovarian abscesses, or systemic illness. This was evident in cases reported by, where delayed or incidental diagnoses led to neonatal urinary retention, sepsis, or abscess formation requiring emergency surgical drainage and nephrectomy. These findings emphasize that OHVIRA, if unrecognized, can present as a surgical emergency in both neonates and adolescents.

Notably, early surgical correction prevents most of these complications. Studies with early intervention strategies, such as researches, reported no postoperative infections, no recurrence of hematocolpos, and complete resolution of pain and obstructive symptoms. These outcomes underscore the critical importance of clinical awareness and prompt intervention to avoid irreversible reproductive morbidity.

**4.**  **Fertility and Obstetric Outcomes: Generally Favorable with Early Correction**

A key concern in managing OHVIRA is the long-term impact on fertility and pregnancy outcomes. Encouragingly, the reviewed literature suggests that timely septum resection preserves reproductive potential in the majority of patients. In a systematic review, reported an 87% pregnancy rate and a 77% live birth rate among OHVIRA patients who underwent septum removal. Other case series confirmed that most patients resumed regular menstruation and conceived without assisted reproduction when intervention occurred early [33].

However, obstetric risks remain elevated. authors documented term and near-term pregnancies in all three of their OHVIRA patients, but each experienced significant complications, including breech presentation, postpartum hemorrhage, and placental retention. Similarly, authors reported two miscarriages followed by a preterm delivery at 33 weeks despite previous adolescent surgical correction. These cases indicate that uterine malformations inherent to OHVIRA, such as didelphys configuration, may predispose to miscarriage, malpresentation, or preterm labor, regardless of prior septum surgery.

Therefore, while fertility is largely preserved, OHVIRA patients should be counseled about potential obstetric complications and offered multidisciplinary antenatal care. Overall, early diagnosis and septum resection optimize outcomes by minimizing pelvic adhesions and endometrial damage, thereby safeguarding the possibility of spontaneous conception and successful pregnancy.

## 4. Discussion

Our four-patient case series reinforces OHVIRA (Herlyn–Werner–Wunderlich) syndrome as a time-sensitive, imaging-driven diagnosis in which timely vaginal decompression and restoration of outflow can prevent secondary sequelae (infection, tubal disease, and endometriosis) while preserving reproductive potential. In our cohort, adolescents presented with cyclical pelvic pain and/or urinary symptoms; ultrasound demonstrated a distended, fluid-filled hemivagina/hematocolpos (Figure 1 and Figure 4), and MRI confirmed uterus didelphys with ipsilateral renal agenesis and the obstructed hemivagina (Figure 2, Figure 5 and Figure 6). Collectively, these findings align with contemporary evidence that OHVIRA is frequently under-recognized at first presentation, with substantial downstream morbidity when diagnosis is delayed.

**Diagnostic delay: why it still happens**

The clinical spectrum observed in our cohort is consistent with contemporary reports showing that OHVIRA presentations range from “typical” post-menarche cyclical pelvic pain with hematocolpos to complicated phenotypes driven by delayed recognition or variant anatomy. In our series, Case 1 presented with fever and foul-smelling discharge, reflecting the infectious sequelae (e.g., pyocolpos/pyosalpinx and pelvic inflammatory changes) increasingly described when obstruction persists. Case 2 demonstrated hematosalpinx complicated by adnexal torsion requiring salpingectomy, paralleling literature that highlights tubal involvement and acute adnexal emergencies as preventable consequences of prolonged outflow obstruction. Case 3 had surgically confirmed endometriosis, supporting the well-described mechanism of retrograde menstruation and the higher endometriosis burden reported in complete obstruction patterns. Finally, Case 4 represents a rare prepubertal complex variant with ectopic/dysplastic ureteral communication—an instructive example of Wolffian duct-associated anomalies that may be incompletely characterized on ultrasound and are more reliably mapped with MRI—thereby reinforcing the need for anatomy-driven, multidisciplinary planning in atypical cases. Even in recent large cohorts, diagnostic delay and misdiagnosis remain common. In a nationwide multicenter study spanning almost a decade, dysmenorrhea and irregular bleeding were prominent symptoms, and complication rates varied by anatomical subtype; incomplete obstruction subtypes were more susceptible to pelvic inflammatory disease, while endometriosis rates were particularly high in complete obstruction patterns [34,35]. Importantly, a dedicated misdiagnosis analysis reported that misdiagnosis is not rare and highlighted system-level contributors, including nonspecific symptoms, incomplete early imaging, and failure to correlate pelvic findings with renal anomalies [36]. These data support a practical message from our series: any adolescent with cyclical pelvic pain, a pelvic mass, or urinary symptoms, especially with known unilateral renal anomaly, should trigger deliberate evaluation for Müllerian obstruction [37].

**Imaging: ultrasound as triage, MRI as the problem-solver**

Our experience mirrors current practice in which ultrasound is a high-value first test, but performance depends on operator expertise and patient anatomy. In our cases, ultrasound reliably identified hematocolpos/obstructed hemivagina (Images 1 and 4), guiding urgent referral. However, ultrasound often cannot fully define the anatomic configuration (uterine duplication, level of obstruction, cervical anatomy, associated tubal disease) or characterize complex variants. MRI, by contrast, provides multiplanar delineation of the uterus, cervices, and vaginas; defines the obstructed compartment; and simultaneously evaluates renal and ureteric anatomy (Images 2, 5, and 6). Recent radiology-focused publications emphasize that embryopathogenesis-aware MRI interpretation improves detection of Wolffian duct-associated variants and helps anticipate concomitant malformations [38]. Likewise, contemporary case-based radiology reports continue to demonstrate MRI’s value when symptoms are atypical or when partial communication between compartments blunts the classic presentation [39]. Taken together, the evidence supports a pragmatic approach: ultrasound as the initial screening tool and MRI for comprehensive anatomical mapping and preoperative planning whenever OHVIRA is suspected [40].

**Surgery: restoring outflow is the cornerstone; approach is individualized**

Across modern series, vaginal septum incision/resection with marsupialization remains the cornerstone intervention, aiming for durable drainage and minimizing the risk of restenosis. In our cohort, hemivaginal septal resection was performed with attention to creating an adequately wide opening and marsupializing the edges; follow-up demonstrated maintained patency and symptom improvement, and postoperative ultrasound in at least one case confirmed complete resolution of hematocolpos (Image 3). Contemporary outcome data in adolescents support these observations: in a recent cohort, standardized septal resection and vaginoplasty were associated with symptom improvement and low recurrence when surgical technique ensured durable patency [41]. In parallel, minimally invasive vaginal approaches are evolving; vaginoscopic or hymen-preserving septal resection has been increasingly reported for virginal adolescents, aligning procedural goals with patient-centered and cultural considerations while maintaining effectiveness [42]. These techniques highlight how surgical planning should be tailored to age, anatomy, and the height/extent of the septum.

**Timing relative to menarche and staged procedures.**

The timing of intervention in OHVIRA should be individualized according to pubertal status, symptom burden, and complications. In post-menarche adolescents with cyclical pelvic pain and obstructed outflow, early definitive septal resection is generally favored once MRI confirms anatomy, as prompt decompression may reduce risks associated with ongoing retrograde menstruation (including endometriosis and tubal damage). A staged approach may be appropriate in selected scenarios: (i) acute infection (e.g., pyocolpos/pyosalpinx) where initial drainage and antibiotic stabilization is required prior to definitive septal surgery; (ii) adnexal emergencies (e.g., torsion/hematosalpinx) where urgent laparoscopy takes priority with subsequent definitive vaginal correction; and (iii) prepubertal or complex urinary-tract variants where multidisciplinary planning (pediatric gynecology/urology) may dictate delayed or stepwise intervention based on symptoms and anatomic risk. In our series, the post-menarche patients were treated in a single stage after MRI mapping, whereas the prepubertal complex variant (Case 4) illustrates that individualized timing and cross-specialty coordination are essential when Wolffian duct–associated anomalies are present.

**The role of laparoscopy: not “routine,” but often decisive**

A key contribution of our series is the pragmatic use of laparoscopy when adnexal pathology is suspected or when complications are likely. In one patient, laparoscopy identified severe tubal disease with torsion and hematosalpinx requiring salpingectomy; in another, endometriotic implants were noted, and postoperative hormonal suppression was instituted. These experiences align with contemporary observations from large cohorts showing that complication profiles differ by subtype and that endometriosis may be particularly frequent when obstruction is complete or longstanding [43]. The literature supports tailoring laparoscopy based on imaging and clinical context rather than using it routinely for every case. For example, laparoscopy-assisted vaginoplasty has been described as successful in selected complex cases, offering improved visualization and the ability to address concomitant pelvic pathology in a single setting [44]. Moreover, endoscopic innovations such as pneumovaginoscopy have been reported for complicated or recurrent infections/abscesses, reinforcing the importance of flexible, individualized surgical strategies in atypical presentations [45].

**Urinary-tract variants beyond renal agenesis.**

Although ipsilateral renal agenesis is the most frequently reported urinary anomaly in OHVIRA, associated Wolffian duct-related variants may extend beyond a “missing kidney” and can be incompletely characterized on ultrasound. These include dysplastic or multicystic renal remnants, ectopic ureteral insertion into the vagina, ureteral dilatation/ureterocele, and contralateral collecting-system changes (e.g., compensatory hypertrophy or pelvicalyceal dilatation). MRI contributes by mapping complex Müllerian anatomy while also clarifying urinary-tract relationships that may alter counseling and operative planning. This was particularly illustrated in our Case 4, in which MRI demonstrated vaginal cystic structures with communication to an ectopic/dysplastic ureter—an anatomic detail not reliably defined by ultrasound alone—supporting the need for MRI-based characterization and multidisciplinary evaluation when urinary tract variants are suspected.

**Implications of the study**

This case series highlights that OHVIRA syndrome should be approached as a radiology-led, time-sensitive diagnosis, where early recognition can prevent avoidable morbidity. Clinically, our findings reinforce that adolescents presenting with cyclical pelvic pain, pelvic mass effect, urinary symptoms, or abnormal menstrual patterns should be evaluated for obstructive Müllerian anomalies, particularly when a unilateral renal anomaly is known or suspected. From an imaging standpoint, our experience supports a pragmatic pathway in which ultrasound serves as the first-line triage tool to detect hematocolpos/hematometrocolpos, while MRI provides definitive anatomical mapping (uterus/cervices/vaginas and urinary tract), identifies complications (e.g., hematosalpinx, inflammatory changes), and directly informs surgical planning. Surgically, the series adds evidence that vaginal septal resection with marsupialization is an effective fertility-sparing intervention with durable symptom relief, while selective laparoscopy is valuable when imaging or clinical findings suggest adnexal complications (e.g., torsion, endometriosis, infection) requiring simultaneous management. Finally, the inclusion of a prepubertal complex variant underscores the need for multidisciplinary coordination (pediatric gynecology, radiology, urology) and supports consideration of screening pelvic imaging in girls with congenital solitary kidney to detect OHVIRA variants before menarche.

**Limitations of the study**

Several limitations should be acknowledged. First, this is a retrospective case series from a single center with a small sample size (*n* = 4), which limits generalizability and precludes any inferential statistical comparison. Second, although the literature synthesis followed a transparent, predefined search and screening strategy with explicit eligibility criteria, the available evidence base for OHVIRA remains dominated by case reports and small case series. As a result, heterogeneity in reporting, imaging protocols, and outcome measures limits quantitative synthesis and precludes formal meta-analysis. These constraints reflect the rarity of the condition rather than methodological weakness and highlight the need for multicenter registries and standardized reporting frameworks. Third, follow-up duration was variable, and one patient was lost to follow-up before intervention, restricting assessment of long-term vaginal patency, recurrence/restenosis, and—critically—reproductive outcomes. Although symptom resolution and anatomical patency after septal resection are considered favorable surrogate outcomes, fertility preservation cannot be inferred from short clinical follow-up alone; therefore, standardized longitudinal follow-up into reproductive age (ideally through the time of attempted conception) is warranted to document menstrual function, recurrence, and fertility/obstetric outcomes in a consistent manner. Fourth, because the study is descriptive, standardized patient-reported outcome measures (e.g., pain scales, quality-of-life instruments) were not uniformly available. Lastly, as a narrative (not systematic) literature review, the synthesis may be subject to selection bias and does not quantify pooled outcomes.

## 5. Conclusions

OHVIRA syndrome is an uncommon but clinically important Müllerian anomaly that often presents with cyclical pelvic pain, obstructive symptoms, and pelvic mass effect in adolescents and may appear in complex or prepubertal forms. Our case series demonstrates that a structured diagnostic pathway, ultrasound for initial detection followed by MRI for definitive anatomical characterization, enables timely diagnosis and appropriate surgical planning. Hemivaginal septal resection with marsupialization provided effective relief of obstruction with maintained vaginal patency and symptom resolution in treated patients, while selective laparoscopy was crucial for identifying and managing associated adnexal complications. Early recognition and intervention are essential to reduce the risks of infection, adnexal damage, and endometriosis, and to optimize long-term reproductive potential.

## Figures and Tables

**Table 1:** Electronic search strategy and keywords used for the literature review (January 2020–December 2025).

Database	Search Strategy (Applied January 2026)	Filters Applied
**PubMed (MEDLINE)**	(“OHVIRA syndrome” OR “Obstructed hemivagina” OR “Herlyn–Werner–Wunderlich syndrome” OR “uterine didelphys with obstructed hemivagina” OR “Müllerian duct anomaly with renal agenesis”) AND (MRI OR “magnetic resonance imaging” OR ultrasound OR ultrasonography) AND (diagnosis OR imaging OR management OR surgery)	English language; Humans; Publication date: 1 January 2020–31 December 2025
**Scopus**	TITLE-ABS-KEY (“OHVIRA syndrome” OR “Herlyn Werner Wunderlich” OR “obstructed hemivagina” OR “uterine didelphys” AND renal) AND TITLE-ABS-KEY (MRI OR ultrasound OR imaging OR diagnosis OR surgical management)	English language; Articles, Reviews; 2020–2025
**Google Scholar**	“OHVIRA syndrome” OR “Herlyn Werner Wunderlich syndrome” OR “obstructed hemivagina renal agenesis” OR “uterine didelphys obstructed hemivagina” MRI ultrasound	English language; 2020–2025; first 200 results screened for relevance

**Table 2:** Clinical Characteristics, Imaging Findings, Management, and Outcomes of Patients with OHVIRA Syndrome.

Case	Age (Years)	Side of Obstructed Hemivagina	Renal Anomaly	Presenting Symptoms	Menstrual History	Key Ultrasound Findings	Key MRI Findings	Surgical Management	Associated Complications	Outcome/Follow-Up
**1**	16	Left	Absent left kidney	Pelvic pain, fever (initially), foul-smelling vaginal discharge	Menarche at 12 years; regular menses, later lighter flow with post- menstrual pain	Vaginal cystic distension	Uterine didelphys; left obstructed hemivagina with fluid collection; absent left kidney	Left hemivaginal septal resection with marsupialization	Pyosalpinx; pelvic inflammatory changes; adhesions	Resolution of discharge and pain; normal menstrual flow; no recurrent obstruction
**2**	15	Left	Absent left kidney	Progressive lower abdominal pain, urinary and bowel symptoms	Menarche at 13 years; initially regular menses, later lighter flow with post-menstrual pain	Markedly distended fluid-filled vagina (hematocolpos)	Uterine didelphys; large hematocolpos compressing bladder and rectum; left hematosalpinx; absent left kidney; mild right pelvicalyceal dilatation	Left hemivaginal septal resection with marsupialization; laparoscopy; left salpingectomy	Hematosalpinx; left tubal torsion	Complete symptom resolution; maintained vaginal patency; normal follow-up ultrasound
**3**	13	Right	Right renal agenesis (solitary left kidney)	Acute right lower quadrant pain, difficulty in urination	Menarche at 11 years; regular menses followed by 6 months amenorrhea with cyclic pain	Markedly distended fluid-filled vagina	Uterine didelphys; obstructed right hemivagina; right renal agenesis	Right hemivaginal septal resection with marsupialization; laparoscopy: Pelvic endometriosis	Mild right hematosalpinx; pelvic endometriosis	Pain resolved; normal menstrual flow; patency maintained on follow-up; treated with hormonal suppression
**4**	11	Left	Congenital solitary kidney with ectopic/dysplastic ureter	Asymptomatic at imaging; history of precocious puberty	Prepubertal (on GnRH agonist therapy)	No hematocolpos; cystic vaginal structures				

**Table 3:** Side-by-side comparison of ultrasound versus MRI findings in the present OHVIRA case series.

Case	Ultrasound: Key Findings (First-Line)	MRI: Incremental Findings (Definitive Mapping)	Practical Impact for Management
1	Vaginal cystic distension	Uterine didelphys; left obstructed hemivagina with fluid collection; absent left kidney	Confirmed anatomy and side of obstruction → planned septal resection + marsupialization
2	Markedly distended fluid-filled vagina (hematocolpos)	Uterine didelphys; large hematocolpos compressing bladder/rectum; left hematosalpinx; absent left kidney; mild right pelvicalyceal dilatation	Defined adnexal complication → septal resection + laparoscopy + salpingectomy
3	Markedly distended fluid-filled vagina (hematocolpos)	Uterine didelphys; right obstructed hemivagina; right renal agenesis	Confirmed OHVIRA side + supported laparoscopy for complications → septal resection; endometriosis treated/managed
4	No hematocolpos; cystic vaginal structures	Uterine didelphys; two left vaginal cystic structures: one communicating with left uterine horn; one communicating with ectopic/dysplastic ureter; no hematocolpos/hematometrocolpos	Identified rare Wolffian-duct variant → multidisciplinary planning (peds gyn/urology)

**Table 4:** Results of the Narrative Literature Review.

Study (Author, Year, Journal)	Study Type	Sample (*N*)	Patient Age(s)	Diagnostic Modality	Surgical Approach	Outcomes	Key Conclusions
Bunnell et al., 2024, *AJP Reports* [27]	Case series (retrospective)	3	Adolescence to adult (diagnosed in youth; pregnancies in 20s–30s)	Ultrasound (initial diagnosis); exam ± laparoscopy for pain/endometriosis	Transvaginal septum excision (vaginoplasty)	All 3 achieved pregnancy after septum resection (one ectopic treated, two term/near-term deliveries); some obstetric complications (preterm labor, malpresentation, retained placenta, etc.) managed	Successful term or near-term pregnancies are possible after early surgical correction, though patients face a wide array of gynecologic and obstetric risks (e.g., miscarriage, malpresentation, preterm birth, PPH) requiring careful management.
Li et al., 2024, *J. Pediatr. Adolesc. Gynecol.* [23]	Case series (retrospective, single-center)	26	Mean ~13.2 years (mostly peripubertal; 92% diagnosed at puberty; one neonatal case)	Pelvic ultrasound (initial evaluation); MRI (preoperative planning—showed uterine didelphys, obstructed vaginal segment, and ipsilateral renal agenesis in all). MRI also assessed septum length and suggested if a postoperative stent might be needed (septum distance >5 cm).	Transvaginal septum resection & vaginoplasty under endoscopic guidance for all except one neonate (managed with temporary transabdominal drain until old enough for surgery). 15% required a postoperative vaginal stent or Foley catheter to maintain patency (all had long septa).	Over median 3-year follow-up, no hematocolpos recurrence or re-obstruction occurred. Dysmenorrhea and pain resolved	A standardized, meticulous surgical approach yields uniformly favorable outcomes in OHVIRA patients, regardless of anatomical variant (side of obstruction, uterine configuration, septum length). Careful preoperative imaging and planning (e.g., anticipating need for stents in long septa) and a systematic septum resection/vaginoplasty protocol resulted in 100% success with no recurrence in this series. This underscores that OHVIRA can be managed definitively with one surgery in most cases, with very low complication and recurrence rates.
Cappello et al., 2018, *BMC Pregnancy Childbirth* [27]	Case report	1	28-year-old female (known OHVIRA from childhood; prior adolescent surgeries)	Prenatal ultrasound (detected ipsilateral renal agenesis, prompting workup in childhood); pelvic MRI (delineated didelphys anatomy and guided surgeries).	Multistage: Adolescent surgeries included removal of a rudimentary hemi-uterus and ipsilateral salpingectomy, plus vaginal septum resection. As an adult, managed with serial monitoring in pregnancy and planned delivery.	Despite earlier surgical correction, she experienced two first-trimester miscarriages. A subsequent pregnancy reached 33 weeks: presented with preterm premature rupture of membranes and breech presentation, delivered via C-section a healthy infant. No recurrent obstruction; fertility was maintained, delivered via C-section a healthy infant. No recurrent obstruction; fertility was maintained, though obstetric course was high-risk (preterm labor).	Even in complex OHVIRA cases with additional anomalies (this patient had limb defects) and prior extensive surgeries, successful pregnancy is achievable. However, patients may face elevated risks of miscarriage and preterm birth. This case highlights the need for meticulous prenatal care and delivery planning in OHVIRA patients. It also demonstrates that prior achievable. However, patients may face elevated risks of miscarriage and preterm birth. This case highlights the need for meticulous prenatal care and delivery planning in OHVIRA patients. It also demonstrates that prior septum resection and uterine-sparing surgery can result in good fertility outcomes (live birth), albeit with potential obstetric complications.
Moufawad et al., 2023, *Gynecol. Minim. Invasive Ther.* [1]	Systematic review (PRISMA-guided)	44 studies	N/A (44 publications; covers pediatric through adult cases)	Various—most cases diagnosed by ultrasound first (screening tool) and confirmed/classified by MRI (to determine septum type and any communication)	Minimally invasive septum removal is first-line (typically transvaginal septum excision). Alternatives described include ultrasound-guided hysteroscopic resection or laparoscopic septum resection; laparotomy is rarely needed. Fertility-sparing surgery is emphasized for reproductive-age patients.	Fertility and obstetric outcomes after proper treatment are generally favorable: e.g., one review of 30 patients showed an 87% pregnancy rate post-septum surgery, with ~77% live birth rate. Early intervention avoids complications (endometriosis, pelvic infection) and allows normal menstruation and pregnancy.	OHVIRA is a complex Mullerian anomaly requiring accurate diagnosis and appropriate management. Minimally invasive, fertility-sparing septum resection is the treatment of choice in most cases. With timely surgery, patients have acceptable fertility and obstetric outcomes, so preserving the uterus is indicated. Early diagnosis/treatment is essential to prevent complications and preserve future fertility.
Sijmons et al., 2023, *Ultrasound (Br. Med. Ultrasound Soc.)* [28]	Case report	1	Newborn (1 day old)	Antenatal ultrasound (flagged right multicystic kidney); postnatal ultrasound (detected intralabial cystic mass → hydrocolpos). MRI was not immediately used due to age; diagnosis confirmed clinically and with ultrasound.	Emergent drainage of hematocolpos via hymenal incision on day 1 of life; IV antibiotics and nephrectomy of the infected dysplastic kidney (within neonatal period). Definitive septum resection to be planned when the child is older (post-puberty) with interim monitoring.	Acute complications were successfully managed: relieved urinary obstruction (anuria resolved post-drainage), prevented urosepsis (by removing the non-functioning infected kidney). The infant recovered; ongoing follow-up is arranged to monitor growth, renal function of the remaining kidney, and to perform septum surgery at puberty.	Early recognition of OHVIRA in the neonatal period prevented serious complications. In this case, identifying the triad (renal anomaly + pelvic mass suggesting duplicated uterus/vagina) allowed prompt intervention—avoiding unnecessary exploratory surgery and averting end-organ damage. The authors stress that when a congenital solitary kidney is detected antenatally, clinicians should screen for Mullerian anomalies early. Timely neonatal intervention (drainage of hydrocolpos) prevented urgent complications (urinary obstruction, infection) and will help preserve the child’s fertility potential long-term.
Yang et al., 2021, *Eur. J. Obstet. Gynecol.* [29]	Case series (retrospective, single-center)	11	3–14 years (pediatric; median 11 years)	Ultrasound (effective first-line; experienced sonographers achieved high diagnostic accuracy); MRI used in select cases for confirmation	Vaginoplasty (resection of obstructing vaginal septum); additional surgeries in select cases (e.g., ureteral reimplantation, nephrectomy for dysplastic kidney)	Follow-up 2 months–8 years (median 5 yrs): relief of obstruction in all; no reported menstrual or fertility issues in those reaching puberty; early surgery avoided infection or endometriosis complications	Early diagnosis (often by ultrasound) and timely septum surgery are crucial. Ultrasound is a highly effective diagnostic tool for OHVIRA, and early surgical intervention prevents genital tract infection and other gynecologic complications, preserving normal menses and future fertility. Clinicians (pediatric surgeons, urologists, gynecologists) should consider OHVIRA in any girl with uterus didelphys or renal agenesis.
Jha & Abdi, 2021, *Clin. Pediatrics* [30]	Case series (3 cases, commentary)	3	10, 13, 15 years (at presentation; one pre-menarche)	Ultrasound (initial discovery of renal/uterine anomalies); MRI (preferred for detailed pelvic anatomy and planning)	Vaginal septum incision/removal (timing individualized: immediate in symptomatic post-menarche cases; delayed until menarche with menstrual suppression in prepubertal case)	All patients had symptom resolution post-septum resection: relief of dysmenorrhea and urinary retention; one prepubertal case monitored until menarche then successfully treated. No re-obstruction reported; potential complications (endometriosis, infection) were averted by timely management.	OHVIRA can present variably (acute urinary retention, chronic pain, or incidental findings), so a high index of suspicion is needed—especially in girls with a solitary kidney. MRI is the gold-standard imaging to delineate anatomy. Prompt septum decompression prevents retrograde menstruation-related endometriosis (reported in 17–35% if untreated) and avoids infections (e.g., pyometra from septal perforation), thereby preserving fertility. Ovarian function remains normal, and fertility is generally good unless compromised by delayed treatment.
Kudela et al., 2021, *J. Pediatr. Urol.* [31]	Case series + systematic review	10 (+734 literature cases)	1 month–16 years in own series (infant to adolescent); literature review included diverse ages	Ultrasound (unilateral renal agenesis often first clue; vaginal distension seen in infants); MRI/VCUG used in some for anatomical details. Focus on identifying anatomical variants (side of obstruction, uterine configuration) in imaging and exam.	Vaginal septectomy (transvaginal resection of septum) in majority (≈87% of reported cases); a minority required more extensive surgery (hemivaginectomy 2%, hemihysterectomy 4%, rarely total hysterectomy <1% for severe variants). ~7.5% of neonatal cases had spontaneous drainage of hydrocolpos requiring no surgery.	Excellent relief of outflow obstruction in most. Endometriosis was found in ~13.6% of cases (in those evaluated laparoscopically)—likely underdiagnosed. No re-obstruction after complete septum removal was noted; infants with spontaneous septum fenestration remained asymptomatic. All patients are advised long-term follow-up for endometriosis risk.	The most common variant of OHVIRA is uterus didelphys with a thick blind hemivagina on the left side and ipsilateral renal agenesis. Vaginal septum resection is sufficient to resolve symptoms and prevent complications in the vast majority of patients. Routine laparoscopy is not required in all cases, but continued monitoring is recommended due to the higher risk of endometriosis—which was present in ~14% of patients and likely underestimated. Early septum surgery effectively prevents such complications.
Zhang et al., 2020, *Gynecol. Endocrinol.* [32]	Case series (imaging study, retrospective)	19	12–34 years (adolescents and adults; median not stated)	MRI (preoperative pelvic MRI for all patients—100% diagnostic accuracy in identifying OHVIRA anatomy). MRI findings: uterus didelphys with double cervices in all; a dilated hemivagina with blood (hematocolpos) on the affected side (T1 hyperintense, T2 dark fluid); absent ipsilateral kidney in all cases (14 right-sided, 5 left-sided).	Hysteroscopic or laparoscopic resection of the oblique vaginal septum in all cases (minimally invasive). No hysterectomies were required; any coexistent pathology (e.g., ovarian cyst, abscess) was addressed during laparoscopy.	All 19 had confirmation of the MRI findings at surgery (septum type and organ anatomy). Symptom relief (resolution of pain and mass effect) was achieved universally after septum removal. Notably, MRI detected associated complications in some cases: 3 patients had ipsilateral ovarian endometriotic cysts, 1 had an ovarian abscess, 1 adenomyosis, 1 ovarian hematoma—these were managed at time of surgery. There were no significant postoperative recurrences reported.	MRI has distinct diagnostic features in OHVIRA and is the preferred modality for evaluation. In this series, preoperative MRI correctly identified the OHVIRA in all patients, including the classification of septum type (16 complete vs. 3 partial) and any concurrent pelvic pathology. MRI clearly showed the triad (didelphys uteri, obstructed hemivagina, absent kidney) and helped plan minimally invasive surgery. The authors conclude that MRI can accurately characterize the variant and complications of OHVIRA, making it the “best and most effective” preoperative imaging tool to guide surgical management. This ensures comprehensive treatment (septum removal and any necessary adjunct procedures) in one stage.
Tong et al., 2014, *Fertil. Steril.* [33]	Case series (single-center, retrospective)	94	Median ~13 years (range not stated; adolescent and young adult women)	Ultrasound + pelvic exam (diagnosis); MRI and diagnostic laparoscopy utilized in select cases. Definitive diagnosis made via surgical findings and pathology.	Vaginal septum resection in all (primary treatment); concurrent laparoscopy to treat endometriosis and lyse adhesions when present (Any associated anomalies like cervical atresia addressed surgically case-by-case.)	Endometriosis occurred in 19% of patients (18/94). All ovarian endometriotic cysts were on the obstructed side. Patients with complete hemivaginal obstruction had a significantly higher endometriosis rate (37%) compared to those with incomplete/partial obstruction (12%). After septum resection, most had resolution of pain and regular menses; no significant long-term fertility impairment noted when treated early.	Unilateral outflow obstruction in OHVIRA strongly predisposes to pelvic endometriosis in adolescents, especially when the hemivagina is completely obstructed. In this large series, about one-fifth of patients had endometriosis due to retrograde menses. Vaginal septum resection is the first-step treatment—it not only resolves the obstruction but also plays a key role in treating/preventing endometriosis and pelvic adhesions. Timely surgery in adolescence is crucial to avoid long-term reproductive morbidity.

## Data Availability

The data presented in this study are available upon request from the corresponding author. The data are not publicly available due to privacy and ethical restrictions.
